# Recent Vaccines against Emerging and Tropical Infectious Diseases

**DOI:** 10.15190/d.2024.6

**Published:** 2024-06-30

**Authors:** Ismail Mazhar, Mir Muhammad Rai, Abdullah Ahmad, Natasha Nadeem, Aamir Shahid Javed, Hassan Mumtaz

**Affiliations:** ^1^Department of Medicine, CMH Lahore Medical College and Institute of Dentistry, Lahore, Pakistan; ^2^Department of Data Analytics, BPP University London, UK

**Keywords:** Vaccines, emerging, tropical, diseases.

## Abstract

Emerging diseases, re-emerging diseases and tropical diseases are a slowly progressing problem globally. This may in part be the result of shifting population, growing poverty, inadequate distribution of resources, or even complacency against personal hygiene. As a result of the low income and low standards of health in developing countries, they provide the perfect breeding grounds for the pathogens and parasites that are the root cause of Neglected Tropical diseases (NTDs). In the case of emerging diseases, most are of zoonotic origin and the recent COVID-19 pandemic is a key example. However, it is not just new diseases but re-emerging diseases such as Influenza that highlight the relentless nature of these infections. Vaccines represent the ultimate safety net against these diseases by bolstering immune systems and lowering subsequent mortality and morbidity of these conditions. In fact, against diseases with high mortalities such as AIDS, Hepatitis, and Malaria, vaccine development has markedly reduced mortality and prolonged life expectancy of those afflicted with these conditions. However, this research highlights the importance of enhancing vaccine efficacy and response. The review further underscores the necessity of research, the timing of vaccine administration, effective resource management by governments, and the perception of the population. Therefore, the review offers valuable insights for the medical community and the pharmaceutical industry in improving research and management to maximize the potential of vaccines.

## SUMMARY

1. Introduction

2. Vaccines, usage, and their effects

3. Vaccines against Neglected Tropical Diseases

4. Vaccines against helminthic NTDs

- 4.1. Soil-transmitted helminthiases - Hookworm, ascariasis, trichuriasis, strongyloidiasis

- 4.2. Schistosomiasis:

- 4.3. Lymphatic filariasis/podoconiosis

- 4.4. Onchocerciasis

- 4.5. Dracunculiasis

- 4.6. Taeniasis/cysticercosis

- 4.7. Echinococcosis

- 4.8. Food - borne Trematodiases - Fascioliasis, clonorchiasis, opisthorchiasis, paragonimiasis

5. Vaccines against protozoal NTDs

- 5.1. Leishmaniasis

- 5.2. Human African trypanosomiasis

- 5.3. American trypanosomiasis

- 5.4. Scabies and other ectoparasites

6. Vaccines against bacterial NTDs

- 6.1. Leprosy

- 6.2. Buruli ulcer

- 6.3. Trachoma

- 6.4. Yaws/other endemic trepenomatoses

7. Vaccines against fungal NTDs

8. Vaccines against emerging diseases

9. EIDs with vaccines undergoing human trials

10. EIDs with vaccines in pre-clinical stage

11. Improvement in policies, practices implementation

12. Conclusion

## 1. Introduction

The emergence and reemergence of old and new infectious diseases are accompanied by the epidemic and pandemic challenges. The emergence and reemergence of diseases are accelerated by factors like rapid human development, and changes in demographics, populations, and the environment. This is also linked to zoonoses in the changing human-animal ecosystem and is affected by a growing globalized society whose geopolitical borders are unrecognized by pathogens. Infectious diseases affect over 1.6 billion people annually, and vaccines are the best prophylactic tool against them^1^. Neglected Tropical Diseases [NTDs] are a group of parasitic and related infectious diseases such as amebiasis, Chagas disease, Cysticercosis, Echinococcosis, Hookworm, Leishmaniasis, and Schistosomiasis, etc ^2^. These diseases are common in low and middle-income countries, living below the World Bank poverty line in conditions prevalent with Human Immunodeficiency Virus (HIV) and Malaria. Additionally, these diseases are highly prevalent in tropical and subtropical regions and are closely associated with poverty, wars, stigma, and marginalized populations who have no access to drinkable, clean water^3^. Despite their global public health importance, progress in developing vaccines for NTD pathogens has lagged due to technical and financial incentives^2^. However, the lack of vaccines against NTDs and EIDs [Emerging Infectious Diseases] is not shocking. Numerous difficulties such as the complex life cycle of some pathogens, low investment in research and lack of industry interest negatively impact the development of vaccines. Another problem is the recent rise of the anti-vaccination movement, which not only impacts public health and vaccination advances but also social and political stability^1^.

Elaborating further, NTDs and EIDs have been documented as being given a lack of attention, giving rise to lack of treatment options and deficient prevention. Tropical diseases impact poverty demographic, representing an unrecognized and major impact of these diseases globally, and are a major hurdle to efforts to enhance human health and reduce poverty.

## 2. Vaccines, usage, and their effects

NTDs are a major threat. These have not been sufficiently discussed or examined regarding their distinctive characteristics. It is important to identify emerging and reemerging neglected tropical diseases. NTDs and emerging and reemerging infectious diseases include HIV, Dengue, Ebola, Chagas disease, Malaria, Leishmaniasis, Zika, and Chikungunya^1^.

EIDs are an additional category that have a major impact on global health. However, EIDs are a result of recently discovered pathogens that have a zoonotic origin while in contrast NTDs comprise mainly of ancient diseases. The theory of coevolution between hosts and pathogens states that eventually, the pathogens develop capabilities to move onto new hosts hence highlighting their sheer unpredictability and the consequent inability to respond to them^4^. This classification first came to global notice in the 1960s with the appearance of viral hemorrhagic fevers, such as Ebola and Crimean-Congo hemorrhagic fever.

Along with NTDs, EIDs also threaten global public health, as they can cause a rise in unpredictable pandemics. Vaccination is one of the most efficient ways to control and prevent infectious diseases. Although technological development has been on the rise in the last few decades, the development of a vaccine that induces a protective and safe immune response is a rather difficult task, especially against most of the NTDs and EIDs^5^. However, the recent advances in vaccine development have allowed the development and licensing of new vaccines that can directly target these diseases^6^.

## 3. Vaccines against Neglected Tropical Diseases

Vaccine development for NTDs has faced many obstacles, the primary challenge remaining the chronic and debilitating nature of the conditions which affect the economic productivity of the region in addition to long-term consequences on human health. As NTDs nearly exclusively occur in the world’s population living in extreme poverty - nearly 700 to 800 million, the complexity of their management “traps” populations in a vicious cycle of poverty^7^. Other barriers in the slow progress of vaccine development have been cited to be greater interest in multi-drug administration programs, lack of funding in Personal Development Planning [PDPs], antigen/adjuvant access discoveries a growing “anti-vax” movement in the world, lack of effective product development and especially, reduced industry interest to invest^7^. Geopolitical challenges are also detrimental, as any initiation of clinical development is impeded by endemic barriers, as there is limited access to standardized equipment, health literacy, human resources, and laboratories in the affected tropics^2^.

Initially, NTD vaccines in the 20th century comprised live attenuated or killed organisms. The difficulty in maintaining living organisms and the costs required, however, has resulted in the favoring of expanding R&D efforts through the use of genomes and proteomes for NTD pathogens. Currently, the stages of vaccine development for NTDs (as classified by the WHO)^8^ have been summarized in Table 1. Only three NTDs have licensed vaccines (dengue, yellow fever, and rabies), while vaccines for the remaining are either in various stages of clinical testing or have had no progress in vaccine development efforts - the “neglected” vaccine development for these remaining NTDs is evident and is further discussed.

## 4. Vaccines against helminthic NTDs

### *4.1. *Soil-transmitted helminthiases - Hookworm, Aascariasis, Trichuriasis, Strongyloidiasis

Compared to other diseases, vaccination efforts in soil-transmitted helminthiases face greater challenges due to their complex life cycles in the environment, avoiding adverse side-effects of vaccines, difficulty in choosing appropriate animal models, and the suitable route of administration of vaccine needed for the immune response^9^. The helminths include Hookworm, Ascariasis, Trichuriasis, and Srongyloidiasis, all in various stages of vaccine development^10^.

**Table 1:** NTDs, Stages of Vaccine Development and Type of Vaccines; Adapted and modified from^2,7,8^

NTD	Stage of vaccine development	Type of Vaccine
Helminths		
Hookworm infection	Phase 1 – 2	Recombinant protein Vaccine
Ascariasis	Preclinical	RNA Vaccine
Trichuriasis	Preclinical	Recombinant protein Vaccine
Strongyloidiasis	–	
Schistosomiasis	Phase 1 – 2	Protein Subunit Vaccine
Lymphatic filariasis/Podoconiosis	–	
Onchocerciasis	Preclinical	Protein Subunit Vaccine
Dracunculiasis	–	
Taeniasis/cysticercosis	Veterinary transmission blocking vaccine	Protein Subunit Vaccine
Echinococcosis	Veterinary transmission blocking vaccine	Protein Subunit Vaccine
Foodborne trematodiases	Preclinical	Protein Subunit Vaccine
Protozoa		
Leishmaniasis	Phase 1 – 2	DNA Vaccine
Human African trypanosomiasis	–	
American trypanosomiasis	Preclinical	Protein Subunit Vaccine
Scabies and other ectoparasites	Preclinical	Recombinant protein Vaccine
Bacteria		
Leprosy	Phase 1	Protein Subunit Vaccine
Buruli ulcer	Preclinical	Protein Subunit Vaccine
Trachoma	Phase 1	Protein Subunit Vaccine
Yaws/other endemic treponematoses	–	
Viruses		
Yellow Fever	Licensed	Live attenuated vaccine
Rabies	Licensed	Inactivated vaccine
Dengue and Chikungunya	Licensed vaccine and additional candidates in clinical development	Live attenuated vaccine
Fungi		
Mycetoma	–	
Chromoblastomycosis and other deep mycoses	–	

### 4.2. Schistosomiasis

The nature of the adult schistosomes is the primary obstacle to vaccine development because of their ability to evade human immune systems, added to by the complex multi-stage cycle and by the potential to elicit allergic reactions^11,12^. For the development of a vaccine, Sm-p80 has been considered the most promising candidate It is postulated that the induction of a balanced immune response by a vaccine candidate would be ideal^13^. However, the resource-limited nature of endemic regions and the aforementioned immunological complexity continue to pose challenges in vaccine development^14^.

### 4.3. Lymphatic filariasis/podoconiosis

There have been no human clinical trials for lymphatic filariasis vaccination underway yet, however, several vaccine candidates have been identified, with the understanding that a vaccine combined with targeted chemotherapy is an optimal approach for the elimination of this disease. Compared to the ease of vaccine studies in animal models in other studies, the life cycle of Wuchereria bancrofti makes it difficult to maintain in rodent models under laboratory conditions^15,16^. Vaccine development attempts include cocktail vaccines, multisubunit vaccines, multi-epitope vaccines, chimeric vaccines, and multivalent vaccines^17^. The nature of the re-emergence of this disease makes it crucial to develop a prophylactic vaccine to eliminate its spread^18^.

### 4.4. Onchocerciasis

Although the global prevalence of onchocerciasis has reduced through mass drug administration (MDA) of Ivermectin, the infection is still difficult to control because Ivermectin cannot be administered in endemic areas co-endemic with loiasis due to the risk of serious adverse effects. Also, ivermectin is not given to children under the age of five, which makes them both vulnerable to infection as well as major reservoirs of transmission^19^. Further, the elimination of onchocerciasis is complicated by the emerging drug resistance to ivermectin^20^. A vaccine is essential to reduce the global burden of onchocerciasis. The Excretory/Secretory products (ESPs) of Onchocerca volvulus have revealed potential vaccine candidates in the form of functional proteins^21^. Thus far, two adjuvanted recombinant antigens, Ov-103 and Ov-RAL-2, have been selected for the development of bivalent vaccines based on consistent results of the induction of protective immunity in mice. The bivalent vaccine is currently in clinical trials in naturally infected cattle and requires further testing in animal models before being forwarded to clinical trials in humans. The utilization of such a vaccine would be vaccination in children under the age of five, which will both prevent infection in children who cannot be given ivermectin, reduce the use of drugs, delay drug resistance, and ultimately, reduce the disease burden as it would reduce adult worm loads.

### 4.5. Dracunculiasis

As of 2022, efforts in Dracunculiasis eradication are only centered around prevention through interventions like prevention of water contamination, and there is no vaccine nor any medication available for the disease^22^.

### 4.6. Taeniasis/cysticercosis

Currently, only a veterinary transmission-blocking vaccine exists in the form of porcine vaccines^23^. There has been no progress for a human vaccine, as animal vaccines remain cheaper. However, calreticulin, a tegument protein of the parasite, has been explored as a potential vaccine candidate, which has demonstrated reduced worm burden in hamster models^24^.

### 4.7. Echinococcosis

A veterinary transmission-blocking vaccine is present for echinococcus. For the development of human vaccines, DNA vaccines with antigen B and recombinant protein vaccines have shown protective immune responses^25^. Additionally, multi-epitope proteins are helpful for immunity against E. granulosus through immunoinformatics approaches. A recombinant leucine aminopeptidase vaccine has been discovered as a potential vaccine antigen of E. multilocularis^26^.

### 4.8. Food-borne Trematodiases - Fascioliasis, Clonorchiasis, Opisthorchiasis, Paragonimiasis

Cathepsin 1 (CL1) is considered the antigen of choice for the development of a diagnostic tool and as a potential vaccine candidate^27^. Studies have been conducted to assess the efficacy of this antigen in F. hepatica and F. Gigantica for the development of a vaccine through trials conducted on animal models in sheep, cows, and goats^28-30^. Further, a chimeric vaccine antigen prepared from FhCatL1 and leucine aminopeptidase (FhLAP) also was demonstrated to elicit an immune response in both rabbit and sheep models^31^. As for F. Gigantica, a study showed the protective efficacy of a combined recombinant vaccine in mice comprising recominant® pro-proteins of cathepsin L1H and B3 (rproFgCatL1H and reproFgCatB3)^32^. A multi-epitope subunit vaccine was also found to be suitable for further investigation for the development of a vaccine against Fasciola Gigantica^33^. However, a meta-analysis found the overall pooled efficacy for all vaccine candidates to be non-significant and indicated further need for testing in animal models^34^.

Candidates for the serodiagnosis of Clonorchiasis have been identified^35^. These can be further studied to test for immune response in animal models. Also, significant worm reduction rates were observed in rats vaccinated orally Bacillus subtilis spores expressing C. sinensis proteins, which has suggested the possibility of preventing clonorchiasis transmission through an oral vaccine in addition to killing cercariea/metacercariae, which are the second intermediate host^35,36^.

There is no current vaccine for Opisthorchis, either. However, successful vaccination has been reported in hamster models using exosome-like extracellular vesicles, recombinant proteins, and chimeric subunit vaccines^37-39^.

Only one study has compared the in-vitro and in-vivo excretory-secretory products for any Paragonimus species (lung fluke), which may indicate targets for the development of vaccines^40^.

## 5. Vaccines against protozoal NTDs

### 5.1. Leishmaniasis

Due to the complicated host-agent interaction, the development of a vaccine is difficult, however, vaccination has promising potential due to the life-long immunity induced by recovery from natural infection^41,42^.

For cutaneous leishmaniasis, five live attenuated vaccines have been explored^43^, and in general, there are Phase 1 and Phase 2 clinical trials underway for the development of a vaccine. Challenges to vaccine development have been stated to be the lack of appropriate adjuvants, which have been discussed in previous literature^44,45^. Progress has also been evident on the exploration of nano-vaccines in previous reviews^46^.

### 5.2. Human African trypanosomiasis

Human African trypanosomiasis has a complex nature which makes it difficult to develop a vaccine. It is documented that Trypanosoma brucei causes loss of immunological memory by disrupting B-cell response and can also evade human immune systems^47^. There is, hence, sparse literature on the development of a vaccine but a multi-epitope vaccine has been formulated^48^.

### 5.3. American trypanosomiasis

A successful vaccine against the parasite is planned to be able to induce type 1 cytokines, cytotoxic T lymphocytes and a lytic antibody response Most studies have tested, in murine models, the efficacy of genes/proteins as prophylactic vaccines^49,50^. The efficacy of α-Gal epitope, Galα13Galβ14GlcNAc bound to human serum albumin (HAS) was examined. C57BL/6 mice immunized with this vaccine elicited anti-α-Gal antibody-mediated humoral reaction and were protected from lethal challenge infection with T. cruzi Y strain (1 × 105 parasite inoculum). A decline was observed in T-cell permeation in tissues, cardiac inflammation, necrotic myocytes and parasite burden in the heart of vaccinated mice^51^. In a recent study, Bivona et al. investigated the 80 kDa prolyl oligopeptidase (Tc80) as a novel immunogen for Chagas vaccine. Mice were immunized with recombinant Salmonella encoding Tc80 (STc80) and recombinant Tc80 protein in a prime-boost approach and displayed that immunized mouse elicited splenic production of Th1 cytokines, such as IFN-γ, IL-2 and TNF-α, Tc80-specific, complement-dependent trypanolytic antibodies, as well as polyfunctional CD4+ T cells and cytotoxic T lymphocytes associated with significant protection from challenge infection and chronic pathology^52^. For the mice infected with T. cruzi Tulahuen cl2 strain, rather than the individual treatments with the vaccine or Bz alone the combined treatment with the Bz and the vaccinenhad a more positive effect on the course of heart disease^51^. For example, delivery of DNA vaccine via the intradermal/electroporation route (vs. intramuscular route) was most effective in generating protective immunity to challenge infection and this vaccine was the simplest in design^49^. This is the first case of the immunogenicity of T. cruzi-derived recombinant antigens formulated as an emulsion with a TLR4 agonist in a non-human primate model. Our results strongly support the need for further exploration of the effect of the vaccine in a therapeutic model of naturally-infected Chagasic non-human primates and evaluation of the preventive efficacy of this type of vaccine, which would strengthen the rationale for the clinical development as a human vaccine against Chagas disease^53^.

### 5.4. Scabies and other ectoparasites

The transcriptomic profile of the parasite and its interactions with a person’s immune system has been studied, therefore helping to increase understanding to support research for vaccine production^54,55^. Currently, an anti-mite vaccine has been tested in mice and a subunit cocktail vaccine has been demonstrated to reduce transmission in rabbits, with more trials needed before the vaccine candidates proceed to clinical stages^56,57^.

## 6. Vaccines against bacterial NTDs

### 6.1. Leprosy

Immunoprophylaxis with the Bacillus Calmette-Guérin [BCG] vaccine is currently the most effective intervention to prevent leprosy^58^. However, the efficacy rates of the BCG vaccine, and with BCG revaccination in combination with chemoprophylaxis not showing very encouraging results for the prevention of leprosy transmission, a Phase 1 clinical trial for the development of a leprosy vaccine is underway comprising a recombinant antigen of Mycobacterium leprae^59^.

### 6.2. Buruli ulcer

Current treatment includes antibiotics, surgery, and the use of BCG vaccine. However, the BCG vaccine’s effects are short-term, and mouse models have been utilized to demonstrate the efficacy of recombinant BCG which expresses the antigenic M. ulcerans proteins^60^. Other proteins like Ag85A have been assessed for potential use in human clinical trials^61^, in addition to work on a multi-epitope vaccine utilizing the PE-PGRS protein through an integrated vaccinomics approach^62^. A study also identified a particular strain of M. ulcerans which could be used to set up immunization studies to test the efficacy of vaccine candidates^63^. Vaccine development remains in pre-clinical stages, with additional R&D required for the development of both diagnostic and preventive tools^64^.

### 6.3. Trachoma

Due to the complex life cycle of Chlamydia, research has extensively explored its molecular pathogenesis to identify potential stages of intervention and proteins of immunogenicity^65,66^. More studies are needed, however, to explore the importance of interferon-gamma production and how it correlates to protection from infection^67^. MOMP is a surface antigen most expressed in Chlamydia, and has been explored as an oral vaccine, with efficacy demonstrated in mice^68^. The most recent progress has been the development of a multivalent vaccine called CTH522 which consists of MOMP proteins and is the only vaccine candidate undergoing Phase 1 clinical trials in humans^69^.

### 6.4. Yaws/other endemic trepenomatoses

The Outer Membrane Proteins [OMPs] of Treponema subspecies can be further studied along with identifying B-cell epitopes to support vaccine development efforts^70^. It is recommended that the vaccine candidate tprL should be studied for vaccination development, and the ability of phase variation resulting in antigenic diversity should be considered^71^.

## 7. Vaccines against fungal NTDs

There has been no vaccine development against fungal infections, let alone fungal NTDs. The use of immunoinformatics tools, and in silico approaches have been recommended to be useful for the development of an efficient fungal vaccine^72^. Given that any development would be directed towards more prevalent fungal infections, it is likely the fungal NTDs will remain neglected for a while yet.

## 8. Vaccines against emerging diseases

Due to the “emerging” nature of this disease, the historical vaccine efforts for these diseases have been hampered by lack of funds and poor market potential, leaving the Global health community vulnerable in times of an outbreak, as seen with the COVID-19 pandemic and Ebola Epidemic^73^. To ameliorate this oversight and bolster global response to future pandemics and epidemics, WHO has released an R&D blueprint for high-priority pathogens that can cause an epidemic^74^. Together with Coalition for Epidemic Preparedness Innovations (CEPI), these efforts have done much to improve the vaccine development for these EIDs, however, it is sobering to realize that the preventive pipeline for this disease still lags behind the efforts for NTDs^75^. The vaccine stages can be seen in Table 2.

**Table 2:** EIDs, Stages of Vaccine Development and Type of Vaccines

EID	Stage of vaccine development	Type of Vaccine
Crimean-Congo Hemorrhagic Fever	Mostly animal models being tested (a Bulgarian vaccine present but it's unlikely to gain international approval)	DNA virus vaccine the most promising in animal models (Bulgarian vaccine is live attenuated)
Ebola Virus	Licensed	rVSV based vaccine
Lassa fever	Phase 1	DNA vaccine
Nipah Virus Fever	Only animal models tested. Vaccine available for horses (Equivac HeV).	Multiple models
Rift Valley Fever	Phase 2	Adenovirus based
Covid-19	Licensed	Multiple licensed

## 9. EIDs with vaccines undergoing human trials

After the coronavirus, Ebola virus, Marburg Virus and Lassa Virus have the vaccines furthest along in development.

Ebola virus is included in WHO’s blueprint for high-priority diseases due to its high mortality rate and the likelihood of person-to-person contact. Before the 2013 outbreak, several vaccine candidates showed promise in small rodent models, but only a few went into the more advanced stages of development. These efforts were also hampered by a dire lack of funds, low market potential, and low cross-protection between viral species^73^. Nonetheless, the 2013 epidemic resulted in increased interest in an efficient vaccine, and many Phase I/II trials were initiated for many different types of vaccines such as a recombinant adenovirus vector (consisting of Ad5.EBOV GP)^76^, replication incompetent modified vaccinia Ankara (MVA) vector^77^ and DNA vaccines^78^.

Recombinant vesicular stomatitis virus (rVSV) showed the most promise with its 2015 Phase III trial giving critical evidence of the utility of a ringed vaccination strategy in an Ebola outbreak^79^. Indeed, even WHO saw VSV as a good candidate platform for vaccines for an outbreak^80^. Subsequently, a rVSV-based vaccine (Ervebo) was FDA-approved and licensed as the first-ever Ebola Vaccine^81^.

Lassa Virus (LAV) vaccine development has seen a similar trajectory as the Ebola virus vaccine. Included in WHO’s high-priority pathogen list due to high mortality rate, lack of therapeutic options and struggles encountered while controlling it during an outbreak^79^, its vaccine efforts have been impeded by its low commercial prospects not offsetting the high cost of vaccine development^82^ and high genetic and biodiversity shown by the virus^83^. Nonetheless, when compared to other EIDs, LAV has one of the most robust development pipelines with many vaccine candidates showing success in animal models and potential for human testing^75^.

WHO has released a rigorous profile for an ideal LAV vaccine which includes elements such as affordability, long-term stability [negating needs for cold chain facilities], protection for special populations [HIV patients, pregnant women, children], efficacy and protection that lasts 3 years[with an option to extend them via boosters] and an uncomplicated method of administration^84^ Several vaccines candidates such as a Measles virus-based vaccine^85^ and an rVSV vaccine^86^, fulfill several of these objectives. From all of the available, a DNA vaccine for Lassa virus was the first to enter Phase 1 of human clinical trials [NCT03805984], with more predicated to follow based on the robust pipeline of Lassa virus and the fact that CEPI has guaranteed funding until phase 2 clinical trials^75^.

Rift Valley Fever Virus (RVFV) is another pathogen added to the WHO blueprint due to its high risk of geographical expansion and high morbidity associated with it^87^. The majority of the vaccine efforts for RVFV have been aimed at Veterinary use^88^. Development of human vaccines has been limited by the strict regulations surrounding human vaccine approval and the need for efficacy studies (which can be difficult for diseases such as RVFV which has intermittent occurrence). Two vaccines have been evaluated for humans; a live attenuated one (MP-12)^89^and a formalin-inactivated (TSI-GSD-200)^90^. Unfortunately, the licensing of these vaccines is limited by safety concerns (MP-12 has shown to be a teratogen in sheep)^91^ and practicality concerns (TSI-GSD-200 needs multiple to be efficacious)^90^. An adenovirus vaccine (ChAdOx1) has shown great potential in animal studies and is expected to enter Phase 1 human trials as well^92^. Considering these ground realities and the fact that RVFV outbreaks in animals precede human epidemics, the current best way to deal with RFVF is to centre your vaccination strategy on animals^88^.

## 10. EIDs with vaccines in pre-clinical stage

Nipah Virus (NiV) is a zoonotic virus that causes respiratory and neurological symptoms in humans (its mortality going up to 80%, such as in the case of the Bangladeshi variant)^93^ causing outbreaks almost annually. Passive immunization options include a recombinant human monoclonal antibody (m102.4) that was found to be safe and well tolerated in a Phase 1 trial^94^. For active immunization, current vaccine vectors, consisting of some of the same vectors as Ebola and Lassa Virus, such as rVSV, Measles, and Adenovirus are under development along with sub-unit vaccines and mRNA vaccines^95^. While many of them have shown great promise in animal trials, none of them have reached the stage of clinical trials in humans, due in part to the complexity of running such trials on a rare disease like Nipah fever^96^. Nonetheless, CEPI now supports several NiV human vaccine candidates, and the recent extensive body of research data can help in the creation of a vaccine that can be licensed in an outbreak. There are also options such as the Equivac^®^ horse vaccine that can help break transmission of the virus to people.

Crimean-Congo hemorrhagic fever virus (CCHFV) is another high-priority pathogen with a global case fatality rate of 40% with higher rates in the developing world^97^. In addition to the problems faced by other EIDs, CCHFV vaccine development is further complicated by the fact that the virus is asymptomatic in most animals. Newborn mice do show signs of the disease, but they are poor models for research because of their immature immune systems^98^. This has been mitigated by the development of humanized mouse models^99^ and cynomolgus macaque models^100^ and they have been a boon in finding suitable vaccine candidates. Just like other EIDs, multiple different vaccine candidates, such as virus vector vaccines, mRNA, DNA, and sub-unit vaccines have shown success in animal models^101^. DNA vaccines have been the first to show results in primates and as such give credence for their use in human trials^102^. Do note that there is a live-attenuated vaccine that has been in use in Bulgaria since the 1960s^103^ but the data on it has been limited and it’s unlikely that this vaccine will ever get global approval due to safety concerns.

**Figure 1:**
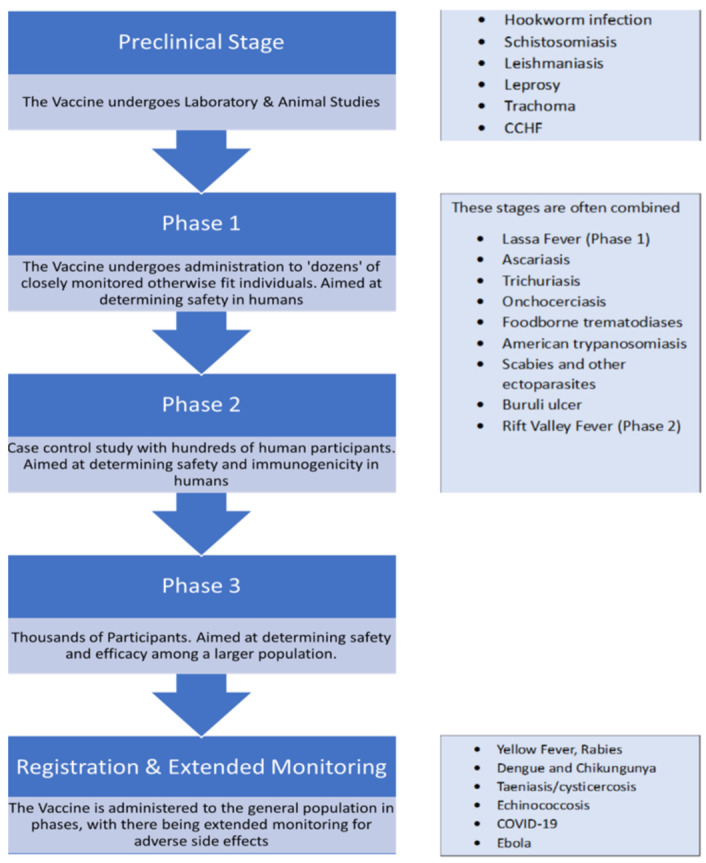
Stages of Vaccine Development

The genetic variability of CCHFV (which results in difficulty creating a single vaccine that could tackle the different virus strains) and the over-reliance on prototype IbAr10200 CCHFV strain (common in ticks but has an unknown virulence status in humans) in most vaccine studies are still huge concerns that must be addressed. The use of heterologous vaccine studies could be one solution^98^. Presently, the stages of vaccine development for EIDs have been summarized in Table 2 and Figure 1.

## 11. Improvement in policies, practices implementation

Neglected tropical diseases (NTDs), have been granted inadequate research due to limited resource allocation, and lead to few interventions. However, new challenges have risen despite the amazing progress made in the fight against neglected tropical diseases. Better drugs, innovative diagnostics and new insecticides are often identified as the priority; however, access to these new tools may not be sufficient to attain and maintain disease elimination, if certain challenges and priorities are not considered. Systemic healthcare policies are needed to eliminate NTDs globally notably in India, China, Russia, Brazil, and South Africa. However, the present policies, practices, and research on them do not meet the need. In terms of policies, attention has been continuously focused on biomedical sciences and excluded ecological, social sciences and interdisciplinary approaches^104^.

Similarly,international collaborations and public-private partnerships have not been focused on systematically. The call from Tungiasis articulated the need a globally scalable collaboration amongst the stakeholders of endemic countries and to develop culturally appropriate communication techniques^105^. Despite marginal improvements in the vaccine and drugs for NTDs, the insufficiency in terms of disease surveillance, control, treatment, prevention, and elimination of NTDs is unmet^106^. A study in Ethiopia provided productive results on implementing an intervention to integrate the diagnosis, reporting, management and prevention of four common NTDs into Ethiopia’s primary healthcare system. The intervention consisted of providing health workers with supportive supervision, adapted job aids, and improved medical and diagnostic supplies. It was implemented for six months, and acceptability and cost-effectiveness were evaluated. Results indicate improvement in detecting, recording and managing target NTDs^107^. Additionally, five steps are suggested for effective policymaking, implementation, and eradication of NTDs which include Community engagement and formalization of community health workers’ role and shift of financial support from disease-oriented programs to availability of donated drugs in health care structures, disease-integrated interventions and improved access to international guidelines for primary health care staff^106^.

In the case of Emerging infectious diseases (EIDs), the problem that further complicates any development of preventative measures is the unpredictability of the pathogens. Moreover, in recent studies, it has been found that the increasingly erratic climate change has led to the increased redistribution of animal species which increases the subsequent risk to the public in those areas. The Intergovernmental Panel on Climate Change (IPCC) has further elaborated on this risk in terms of climate-related hazards and vulnerability of human and ecological systems. To meet this evolving challenge of EIDs, it has become vital to form a unified approach that addresses human, environmental, and animal health together and not the previous single-aim approach that barely yielded results. Hence the aptly coined “One Health Approach”^27^. The ‘One Health Approach’ was first named in 2003-2004 and was associated with the 2003 emergence of SARS and the emergence of a particularly virulent strain of avian flu. To elaborate, the One Health Approach focuses on the response and action at the human-animal-ecological interface, especially keeping in light the zoonotic diseases. Additionally, the core of this approach is interdisciplinary collaboration and is envisioned by numerous organizations such as WHO and UNICEF to also include other fields such as biodiversity, social sciences, and ecology^28^.

Advances in vaccine development have resulted in a variety of new vaccines being introduced into recommended immunization schedules. Armenia introduced the the pneumococcal conjugate vaccine (PCV) and rotavirus vaccine (RV). Multilevel logistic regression models were used to evaluate community- and individual-level factors associated with uptake. When developing strategies for new vaccine implementation, characteristics of the patient such as residence, distance to health clinics, age and siblings must be considered. Further exploration of cluster differences may provide better evaluation based on the increased uptake of these and other new vaccines^16^.

A recurring issue is the unpredictability of the outbreaks of any emerging infectious disease. Consequently, this makes it difficult to stockpile vaccines for extended amounts of time as they have short shelf lives and even if some can be preserved by methods such as cold chains, the required infrastructure is often extremely challenging to manage in developing countries where these diseases are most prone to emerge. However, the development of mRNA vaccines has rendered these concerns obsolete, owing to how quickly they can be manufactured, and the costs subsequently lowered by employing self-amplifying RNA. This solution was especially proven during the COVID-19 pandemic where rapid development of mRNA vaccines provided the safety blanket required by the global population to return to normalcy once again. Thus, the development of such types of vaccines may prove to be the solution for further rapid responses to any new EID^29^.

## 12. Conclusion

It is concluded that vaccines are undeniably effective in providing a crucial safety net against emerging, reemerging, and tropical diseases by strengthening immune systems, reducing mortality, and extending the life expectancy of affected individuals. However, the effectiveness and response of the vaccines depend on the extent of research, the timing of administration, governmental resource management, and public perception. Therefore, it is further concluded that reform and proper management are essential to fully realize the potential of vaccines. The review is likely to benefit medical community and pharmaceutical industry in aligning to latest research and management of resources to maximize the benefits of vaccines.
